# A validation for sex differences in gut microbiome of essential hypertension based on cohort analysis

**DOI:** 10.1186/s12866-025-04500-8

**Published:** 2026-02-07

**Authors:** Pan Wang, Yaxuan Yao, Kaixin Yan, Siyuan Wang, Minjie Wang, Xiaoyan Liu, Chaowei Hu, Ying Dong, Jing Li

**Affiliations:** 1 Heart Center and Beijing Key Laboratory of Hypertension, Beijing Chaoyang Hospital, Capital Medical University, Beijing, 100020 China; 2 Department of Cardiology, Beijing Chaoyang Hospital, Capital Medical University, Beijing, 100020 China; 3 Beijing Anzhen Hospital, Capital Medical University, Beijing Institute of Heart, Lung and Blood Vessel Diseases, Beijing, 100029 China

**Keywords:** Hypertension, Sex, Gut, Microbiome

## Abstract

**Background:**

Prior research has demonstrated sex-specific differences in hypertension (HTN). The gut microbiota (GM) and its metabolic functions have emerged as key players in the development of HTN. To explore potential sex-based heterogeneity in gut bacteria among hypertensive patients, we conducted this study with the aim of validating sex differences in the gut flora associated with HTN.

**Methods:**

Here, we leveraged a metagenomic dataset comprising 106 fecal samples from a Chinese cohort of individuals with essential HTN to systematically analyze and compare alterations in the gut microbiome between male and female patients, as well as relative to a healthy control group.

**Results:**

Our study confirmed a statistically significant difference in the β-diversity of GM between hypertensive patients and healthy controls. When the subjects were further stratified by sex, significant differences in the distribution of gut flora were observed exclusively in females, whereas none was noted between groups in males. It was observed that certain genera of GM exhibit negative correlations with blood pressure. Notably, the relative abundance of these bacterial genera, including *Lachnospir*a, *Faecalibacterium*, and *Roseburia*, was significantly diminished in female hypertensive patients. These organisms are primarily involved in the biosynthesis of short-chain fatty acids (SCFAs), with a notable emphasis on butyrate production. *Ruminococcus gnavus* was specifically enriched in hypertensive males, whereas certain bacteria, such as *Lactobacillus*, were notably depleted. The abnormality of the SCFAs-producing flora in female hypertensive patients may be related to that women are more likely to develop hypertensive organ damage.

**Conclusions:**

The findings of our study indicate that GM dysbiosis is more significantly associated with HTN in females. Consequently, sex constitutes a critical factor in evaluating the role of intestinal flora in the pathogenesis of HTN.

**Supplementary Information:**

The online version contains supplementary material available at 10.1186/s12866-025-04500-8.

## Introduction

HTN stands as one of the foremost risk factors for global mortality. In the past three decades, the prevalence of HTN has more than doubled on a global scale, with a particularly pronounced increase in low- and middle-income countries. Uncontrolled HTN can result in serious complications such as stroke, ischemic heart disease, heart failure, chronic kidney disease, vascular dementia, and other severe health conditions [1]. Epidemiological studies have demonstrated that HTN resulted in millions of deaths annually due to adverse cardiovascular events. In 2019 alone, it was responsible for 10.8 million fatalities [2, 3]. Currently, blood pressure management in hypertensive patients continues to present a significant challenge. Only approximately half of the affected population has received antihypertensive therapy, and merely one-third are able to maintain their blood pressure within recommended parameters [4].

Since the 1940 s, sex differences in blood pressure have been acknowledged [5]. There are significant disparities between males and females in various aspects of HTN, including pathophysiology, prevalence and awareness, optimal blood pressure targets, efficacy and safety of antihypertensive medications, and ideal dosing regimens [6–10]. Despite the pathophysiologic mechanisms not being fully elucidated, they likely encompass a diverse array of factors, notably sex-related vascular alterations. These include endothelial dysfunction, diminished vascular compliance, augmented arterial stiffness, and variations in blood pressure regulation mechanisms. The latter may involve changes in sympathetic tone, activity of the renin–angiotensin–aldosterone system, levels of nitric oxide, endothelin-1, bradykinin, and natriuretic peptides, as well as differences in renal salt handling capacity [11]. Furthermore, the adverse consequences of HTN exhibit sex-specific heterogeneity. Female patients tend to develop hypertensive organ damage at an earlier age and even at relatively lower blood pressure levels compared to their male counterparts [5]. A recent sex-pooled analysis of several studies, including the Framingham Heart Study, the Multi-Ethnic Study of Atherosclerosis, the Atherosclerosis Risk in Communities Study, and the Coronary Artery Risk Development in Young Adults Study, revealed that women experience a greater increase in blood pressure compared to men. This disparity persists from the age of 30 throughout the entire lifespan. The early sex difference may underlie the divergent cardiovascular disease (CVD) risks observed between women and men later in life [12].

In recent years, a growing body of evidence from both animal and human studies has demonstrated that the GM and its metabolites play a pivotal role in the development and pathogenesis of HTN [13–16]. Abnormalities in the structure of the GM have been observed in various animal models and hypertensive patients, characterized by reduced microbial diversity and structural and functional impairments [17–19]. Previous study revealed a significant increase in blood pressure in germ-free mice following the transplantation of GM from hypertensive patients. This finding provides direct evidence of the pathogenic role of intestinal dysbiosis in HTN [17]. Furthermore, it has been reported that gut-derived metabolites such as SCFAs, along with prebiotics and probiotics, possess the potential to modulate both systolic and diastolic blood pressure, which further underscores the critical role of the GM in blood pressure regulation [20, 21].

The microbial composition is known to be influenced by a multitude of factors, including genotype, dietary habits, age, ethnicity, geographic location, and the host's health status, and it also demonstrates variability based on sex Studies have indicated that females exhibit a higher ratio of Thick-walled bacilli to Anamorphic bacilli compared to males [22]. During puberty, GM start to diverge between males and females, a process influenced by factors including sex hormones, dietary patterns, metabolic conditions, and inflammatory states. Alterations in GM characteristics can in turn influence inflammation levels, metabolic states, and sex hormone homeostasis, which play a role in the pathophysiological mechanisms of diseases such as obesity, autoimmune disorders, polycystic ovary syndrome, and arterial stiffness [23]. Therefore, it was wondered whether sex-based heterogeneity exist in the GM of hypertensive patients. Recent studies indicate that there are notable sex-specific differences in GM among patients diagnosed with HTN through ambulatory blood pressure monitoring. Specifically, significant disparities in β-diversity and gut microbiome composition were observed between the HTN and HC groups, with this dysbiosis being evident exclusively in female subjects [24]. This indicates that gut flora dysbiosis may be more significantly associated with female HTN, potentially mediated through circulating metabolites. However, the sex-specific differences in the association between essential HTN and GM require validation through external cohort studies.

The present study utilized metagenomic data from a Chinese cohort of primary HTN patients stratified by sex, and conducted an in-depth analysis of microbial diversity and the characteristics of differentially abundant bacteria, aiming at further validating the sex-based heterogeneity in the gut microbiome of hypertensive individuals.

## Methods

### Study cohort

The publicly available metagenomic sequence dataset utilized in this study originates from a previously reported cohort study on HTN in China [25]. The dataset is archived in the European Bioinformatics Institute database under the accession number ERP023883. In this study, a total of 106 participants diagnosed with essential HTN and HCs were recruited. Participants were stratified into four groups based on their blood pressure levels and sex: female hypertensive patients (female, systolic blood pressure ≥ 140 mm Hg or diastolic blood pressure ≥ 90 mm Hg, *n* = 22, HTN-F), male hypertensive patients (male, systolic blood pressure ≥ 140 mm Hg or diastolic blood pressure ≥ 90 mm Hg, *n* = 34, HTN-M), female healthy controls (female, systolic blood pressure ≤ 120 mm Hg and diastolic blood pressure ≤ 80 mm Hg, *n* = 23, HC-F), and male healthy controls (male, systolic blood pressure ≤ 120 mm Hg and diastolic blood pressure ≤ 80 mm Hg, *n* = 27, HC-M). Exclusion criteria comprised individuals with a diagnosis of cancer, chronic inflammatory conditions, severe psychiatric disorders, other significant medical conditions, a history of substance dependence, or a history of antibiotics or anti-inflammatory in the recent 2 months before sampling. Subjects were also excluded if they had symptoms of respiratory infection or digestive tract disease. Additionally, participants with HTN or severe cardiovascular diseases (such as, coronary artery disease or stroke) history in recent 5 years were excluded for healthy controls. We matched other factors such as age, BMI, and smoking status between HTN and HC groups to minimize the effect on our findings.

### DNA extraction and metagenomics sequencing

Approximately 5 ml of fresh fecal samples collected during a single bowel movement, obtained from enrolled subjects via natural voiding, were promptly collected and transferred to sterile cryogenic tubes. The samples were then stored at −80 °C for subsequent processing. DNA was extracted from the fecal samples following established protocols [26]. The DNA concentration was quantified using a NanoDrop spectrophotometer (Thermo Scientific, Waltham, MA, USA), and the quality was evaluated through agarose gel electrophoresis. All extracted DNA samples were subsequently sequenced using an Illumina HiSeq3000 sequencer.

DNA libraries with an insert size of 300 bp were constructed, and paired-end sequencing was performed to generate 100 bp reads in both forward and reverse directions. Upon completion of the sequencing process, a series of bioinformatics analyses were conducted as the following detailed description, including encompassing gene catalog construction, gene prediction, taxonomic annotation, and relative abundance estimation.

### Gene catalogue construction and taxonomic annotation of metagenomic data

We utilized the Cluster Database at High Identity with Tolerance (CD-HIT, version 4.5.8, La Jolla, California, USA) to construct non-redundant gene libraries. The clustering procedure was conducted with a sequence identity threshold set at 95% and a minimum alignment coverage of 90%. About gene prediction, open reading frame (ORF) prediction was performed on assembled contigs using MetaGeneMark (version 2.10), a gene prediction software specifically optimized for prokaryotic sequences in metagenomic data [27]. MetaGeneMark utilizes statistical models trained on known microbial genomes to accurately identify coding regions. Taxonomic assignment of predicted genes was conducted by aligning the sequences against the NCBI non-redundant protein (NR) database using DIAMOND (version 0.7.9.58), an accelerated alignment tool for high-throughput metagenomic data. The alignment was performed using the following parameters: a maximum of 50 target hits per query (-k 50) and a sensitivity threshold of --sensitive -e 0.00001. Hits with e-values ≤ 10 were retained for taxonomic classification. The lowest common ancestor (LCA) algorithm implemented in MEGAN (MEtaGenome ANalyzer) was then applied to assign taxonomic ranks based on the DIAMOND alignment results. Taxon-level abundance was calculated as the cumulative sum of abundances of all genes assigned to each taxon. To estimate the relative abundance of gene and species, reads were mapped to the non-redundant gene catalog using SOAP2, a high-performance short-read aligner. The alignment was executed with optimized parameters (-m200—× 400 -s119) to enhance mapping precision. Genes with fewer than three mapped reads were filtered out to ensure statistical robustness. Gene abundance was normalized based on both read counts and gene lengths, and the relative abundance of each taxon was subsequently calculated by aggregating the normalized abundances of its corresponding genes.

### Microbial diversity

To evaluate the microbial diversity of gut bacteria in patients with essential HTN and HCs, we employed a series of analytical methods. Specifically, we utilized the vegan package (version 2.6.4) within R software (version 3.6.3) to calculate several α-diversity indices based on species abundance. The Shannon index was calculated based on the relative gene abundance to demonstrate the within-sample diversity. The relative abundance of gene in each sample were estimated by dividing the number of reads uniquely mapped to that gene by the length of gene region and by the total number of reads from the sample. These indices included the observed richness, Chao1 richness estimator, ACE richness estimator, Simpson diversity index, and Pielou's evenness index, providing a comprehensive characterization of the gut bacterial diversity within the samples. Additionally, to assess the statistical significance of differences in α-diversity metrics between samples with varying health statuses, we employed the Wilcoxon rank-sum test test to get the raw p-values. Then we applied the Benjamini–Hochberg method for multiple testing correction to the raw p-values in order to increase the rigor of the results. We further conducted an analysis of β-diversity to quantify the differences among various microbial communities. Specifically, Principal component analysis (PCA) was conducted based on Bray–Curtis distance using the FactoMineR and ggplot2 packages in R. The Analysis of Similarities (Anosim) was calculated by vegan package in R to test the significance of the differences between groups in terms of β-diversity. Principal Coordinate Analysis (PCoA) was performed based on the Bray–Curtis dissimilarity index, and β-diversity differences were visualized using the vegan package in R software. Nonmetric Dimensional Scaling (NMDS) was conducted using the vegan package within the R software (version 3.3.3). Linear discriminant analysis (LDA) Effect Size (LEfSe) analysis was then performed to identify microbial features that were significantly differentially abundant between male and female cohorts, respectively [28]. Features with differential abundance across groups were first selected by Kruskal–Wallis test (*p <* 0.05), followed by pairwise Wilcoxon tests to ensure consistent inter-group differences. Biological effect sizes were estimated using Linear Discriminant Analysis (LDA), with LDA Score > 2 considered significant. The results were visualized using evolutionary branching diagrams (cladograms) and histograms. Another differential abundance testing was also employed to increase the completeness and accuracy of the results. Specifically, ANCOM-BC2 fits a bias-corrected log-linear regression framework to microbial count data, explicitly accounting for compositionality, overdispersion, and excess zeros [29]. Taxonomic abundance tables at the species level were used as input, with raw counts retained to allow model-based bias correction. Before model fitting, low-prevalence taxa were filtered to reduce data sparsity and improve statistical power. Parameter prv-cut was setted as 0.1, meaning that only taxa present in at least 10% of the samples were retained. In addition, the parameter lib-cut was set as 0 to retain all samples regardless of sequencing depth. Differential abundance testing was based on Wald statistics, and multiple testing correction was performed using the Benjamini–Hochberg procedure to control the false discovery rate. Taxa with an adjusted p-value < 0.05 were considered significantly differentially abundant. All analyses were performed by the ANCOMBC package in the R software (version 4.5.1).

### Statistical analysis

Continuous variables were presented as mean ± standard deviation (SD) or median [interquartile range: first to third quartiles] and analyzed using Student's t-test or the Wilcoxon rank-sum test, while categorical variables were reported as frequencies (percentages) and evaluated using the chi-squared test. All data analyses were conducted using SAS version 9.3 (SAS Institute Inc., Cary, NC, USA). Two-tailed p-values were calculated, with a threshold of *p <* 0.05 for determining statistically significant differences. Benjamini–Hochberg method was employed for multiple testing correction to the raw p-values in order to increase the rigor of the results. Spearman correlation analysis was employed to evaluate the correlation between species, with a cutoff for the correlation coefficient set at ≥ 0.2 or ≤ −0.2, and all p-values were less than 0.05. Subsequently, we utilized Spearman correlation analysis to assess the degree of correlation between the top 30 most divergent species in abundance between groups and various clinical indicators. To visualize the correlation networks and heat maps, we utilized the OmicStudio platform (https://www.omicstudio.cn/tool) and the igraph package (version 1.2.6) within the R environment [30, 31].

## Results

### Demographic and clinical characteristics of the study cohort

In this study, a total of 22 hypertensive females (HTN-F), 34 hypertensive males (HTN-M), 23 healthy control females (HC-F), and 27 healthy control males (HC-M) were recruited. HTN was defined according to the medical criteria of a systolic blood pressure (SBP) of 140 mmHg or higher, or a diastolic blood pressure (DBP) of 90 mmHg or higher. Table 1 provides a detailed summary of the clinical characteristics of all participants. Both SBP and DBP were significantly elevated in the HTN group compared to the HC group of the corresponding sex. No statistically significant differences were observed in other clinical biochemical parameters, including body mass index, fasting blood glucose levels, total cholesterol levels, triglyceride levels, low-density lipoprotein cholesterol, and high-density lipoprotein cholesterol.

**Table 1:** Baseline Characteristics of Study Participants

Characteristics	HTN-F	HTN-M	HC-F	HC-M	p_1_-Value (HTN-F vs HTN-M)	p_2_-Value (HTN-F vs HC-F)	p_3_-Value (HTN-M vs HC-M)	p_4_-Value (HC-F vs HC-M)
Number	22	34	23	27	/	/	/	/
Age, years	60.50(50.25–68.00)	54.00(47.75–63.00)	55.00(48.00–59.00)	54.00(50.00–62.00)	0.190	0.117	0.816	0.668
Systolic pressure, mm Hg	165.00(150.00–181.25)	160.00(144.00–180.00)	112.00(110.00–118.00)	110.00(110.00–116.00)	0.239	< 0.001	< 0.001	0.506
Diastolic pressure, mm Hg	100.00(91.50–110.00)	97.50(90.00–107.75)	70.00(66.00–78.00)	70.00(65.00–76.00)	0.416	< 0.001	< 0.001	0.977
Height, cm	161.50(155.00–169.25)	164.00(158.75–169.25)	158.00(152.00–165.00)	164.00(156.00–170.00)	0.416	0.151	0.636	0.073
Weight, kg	61.50(57.50–66.25)	65.00(55.00–70.25)	58.00(51.00–71.00)	62.00(50.00–67.00)	0.322	0.375	0.219	0.539
Body mass index, kg/m^2^	23.25(21.40–25.40)	24.60(20.60–25.85)	23.70(21.30–25.80)	23.10(21.10–24.90)	0.314	0.768	0.437	0.579
Fasting blood glucose, mmol/L	5.76(4.67–8.38)	5.56(4.91–7.68)	5.23(4.76–6.60)	5.66(4.92–7.83)	0.609	0.329	0.873	0.316
High density lipoprotein, mmol/L	1.11(1.03–1.28)	1.14(0.96–1.33)	1.17(1.01–1.31)	1.20(1.00–1.44)	0.880	0.525	0.194	0.647
Low density lipoprotein, mmol/L	3.20(2.43–3.75)	3.04(2.60–3.38)	3.06(2.36–3.83)	2.88(2.52–3.29)	0.906	0.973	0.523	0.647
Triglyceride, mmol/L	1.60(1.18–2.52)	1.74(1.28–2.63)	1.72(1.26–2.08)	1.31(0.68–2.08)	0.731	0.768	0.026	0.227
Total cholesterol, mmol/L	5.12(4.67–5.55)	4.92(4.31–5.54)	5.24(4.13–5.93)	4.95(4.29–5.58)	0.669	0.768	0.942	0.661
Smoking (Y/N)	5/17	13/21	5/18	15/12	0.257	1.000	0.205	0.021
Diabetes (Y/N)	9/13	10/24	6/17	11/16	0.401	0.353	0.422	0.372

p_1_ value: HTN-F versus HTN-M, p_2_ value: HTN-F versus HC-F, p_3_ value: HTN-M versus HC-M, p_4_ value: HC-F versus HC-M, HTN-F: Female with HTN, HTN-M: Male with HTN, HC-F: Female health control group, HC-M: Male health control group

### Diversity of intestinal flora

**Fig. 1:**
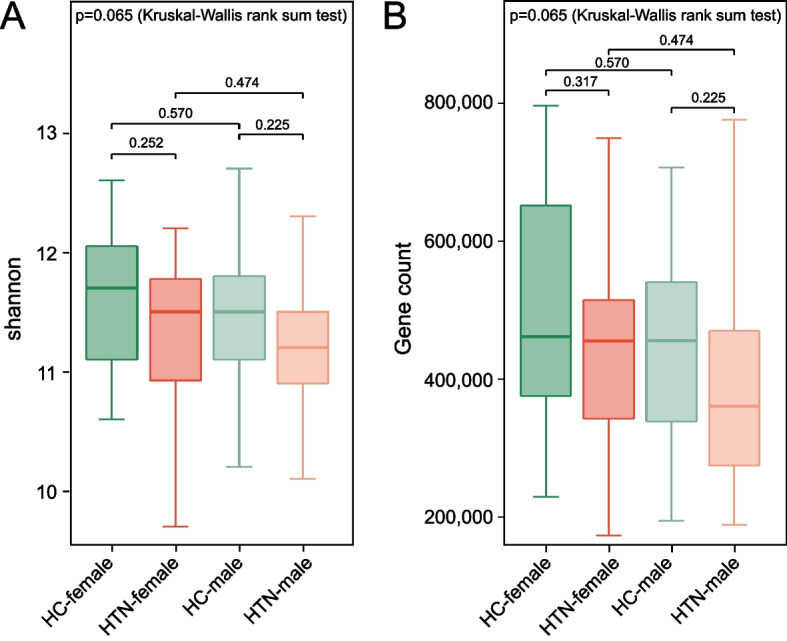
Comparison of the shannon index and gene count of GM between HTN and HC individuals, stratified by sex. **A** The Shannon index, which reflects α-diversity at the species level for each group: HC-females (HC-F, *n* = 23), HC-males (HC-M, *n* = 27), HTN-females (HTN-F, *n* = 22), and HTN-males (HTN-M, *n* = 34). **B** Gene count of GM in each group. P-values were calculated by Wilcoxon rank-sum test and then adjusted by Benjamini–Hochberg method for multiple testing

**Fig. 2:**
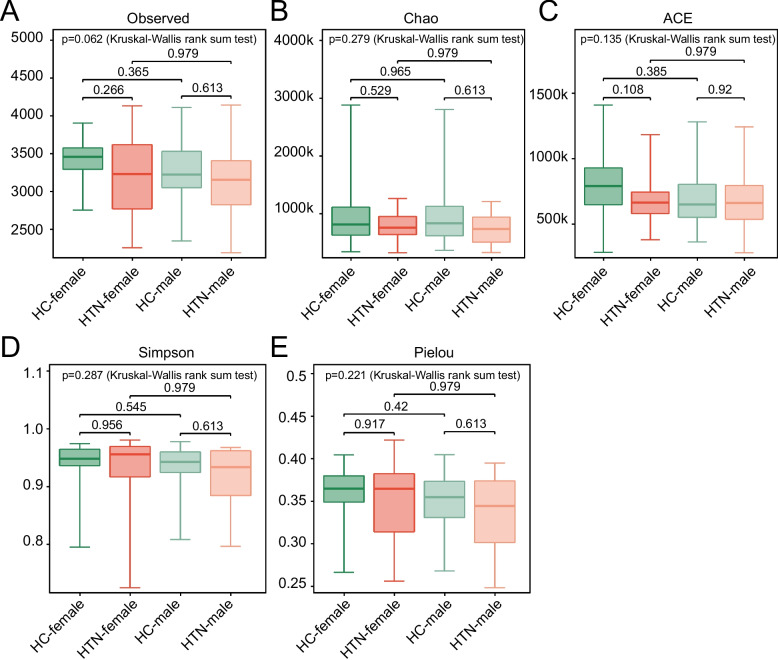
Alterations in gut microbial α-diversity among hypertensive individuals compared to HCs, stratified by sex at the species level. **A** Species richness in each group. **B** Chao richness index. **C** ACE index, with statistically significant differences observed between the HTN-F and HC-F groups. **D** Simpson diversity index. **E** Pielou's evenness index. P-values were calculated by Wilcoxon rank-sum test and then adjusted by Benjamini–Hochberg method for multiple testing

For the analysis of α-diversity, we employed a comprehensive set of indices to evaluate the GM structure in HTN-M, HTN-F, HC-M, and HC-F. These indices included the Shannon diversity index, gene count, observed species richness, Chao1 richness estimator, ACE estimator, Simpson index, and Pielou's evenness index. Although slight variations in the Shannon index and ACE estimator were noted between HTN-F and HC-F at the species level (Fig. 1A, C), these differences did not reach statistical significance. And gene counts showed comparable variation in the female groups (Fig. 1B). Results based on these α-diversity indices revealed no obvious discrepancy between HTN-M and HC-M groups across all examined metrics (Figs. 1, 2).

**Fig. 3:**
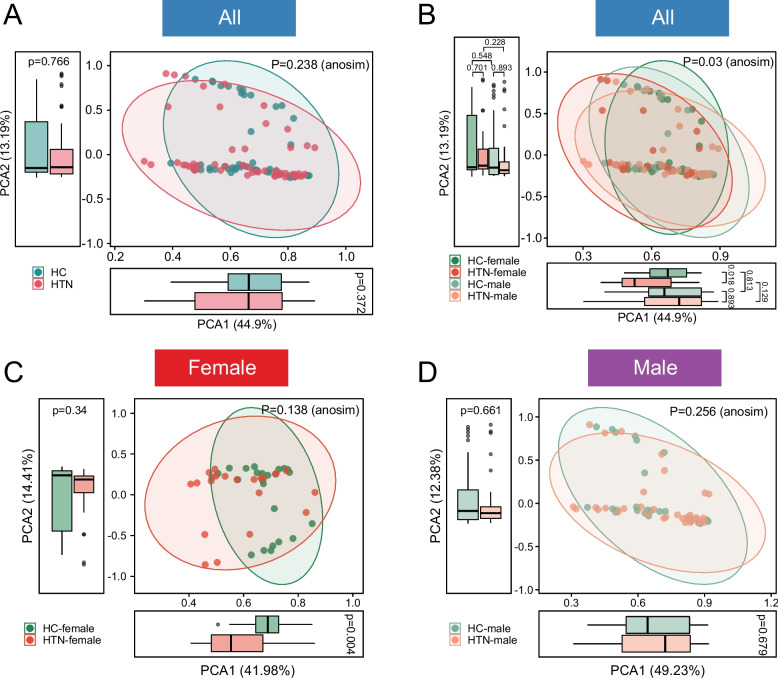
Characterization of β-diversity of GM in HTN patients of different sex s by PCA. **A** PCA was conducted at the species level for both the HC and HTN groups. **B** At the species level, PCA distribution was analyzed across four subgroups: HTN-female (HTN-F), HTN-male (HTN-M), HC-female (HC-F), and HC-male (HC-M). Scatter plots revealed significant differences in PCA distribution among these four groups. Box plots highlighted a statistically significant difference between HTN-F and HC-F along the PCA1 axis. **C**-**D** β-diversity based on PCA was compared between HTNs and HCs, stratified by sex. Box plots indicated a significant difference in PCA1 distribution between HTN-F and HC-F, whereas no such difference was observed between HTN-M and HC-M. P-values were calculated by Wilcoxon rank-sum test and then adjusted by Benjamini–Hochberg method for multiple testing

**Fig. 4:**
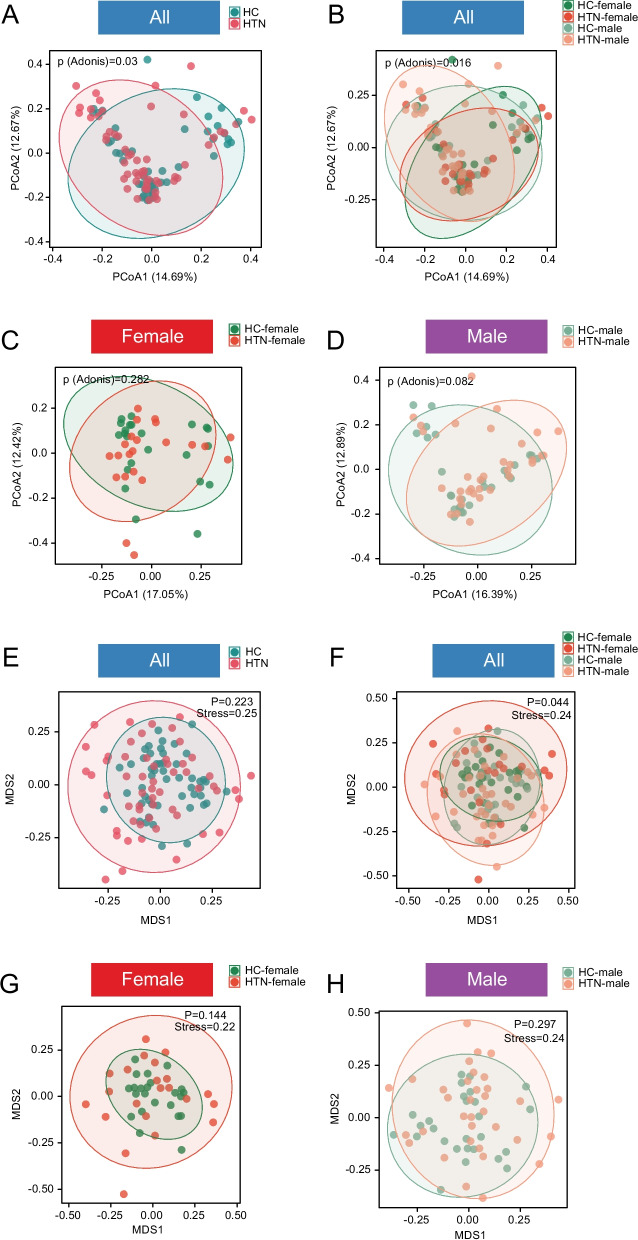
PCoA and NMDS were employed to illustrate the β-diversity of GM in HTN. **A** PCoA comparison between HTN and HC revealed statistically significant differences at the species level. **B** Further analysis of PCoA was conducted across four subgroups: HTN-F, HTN-M, HC-M, and HC-F. The plots indicated significant variations in the distribution among groups. **C**-**D** PCoA was separately compared between HTN and HC for each sex. **E** NMDS scatter plots demonstrated the differences between HTN and HC. **F** NMDS analysis revealed significant disparities among HTN-F, HTN-M, HC-M, and HC-F groups. **G**-**H**, The diversity based on NMDS in hypertensive and normotensive control groups was analyzed with consideration of sex-specific differences

Therefore, we subsequently assessed the β-diversity of the GM community through PCA, PCoA, and NMDS analyses. When comparing the HTN group with the HC group irrespective of sex, PCA failed to effectively differentiate and cluster these two groups, as illustrated in Fig. 3A. Upon stratification of the HTN and HC groups by sex, significant differences were observed in the microbial communities of the GM (Fig. 3B). Notably, in the female subgroup, the PCA analysis revealed a statistically significant difference between HTN-F and HC-F samples along the first principal component axis (Fig. 3C), a distinction that was not evident in the male subgroup (Fig. 3D). PCoA further confirmed significant differences in GM structure among the four groups, comprising male and female hypertensive patients and HCs. However, no significant differences were observed within each sex group (Fig. 4A-D). The NMDS analyses further substantiated this finding, revealing trends that are in alignment with the aforementioned results (Fig. 4E-H). In conclusion, our study demonstrates that the GM structural characteristics of HTNs and HCs were comparatively more similar in males, whereas significant differences were observed between groups in females.

### Disparities in the composition of gut microbiome between groups

**Fig. 5:**
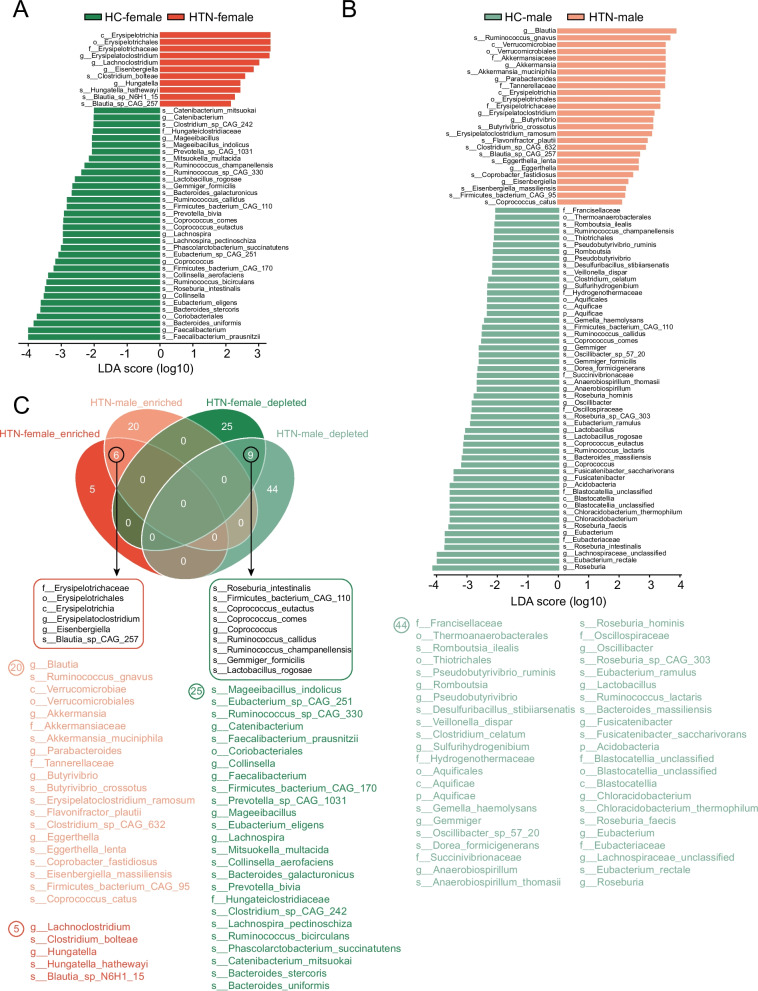
A sex-based analysis of taxa exhibiting significant differences between HTN and HC groups. **A** Linear Discriminant Analysis (LDA) score bar graphs highlighting taxa with statistically significant differences between HTN-F and HC-F. **B** LDA scores for bacteria showing significant differences between HTN-M and HC-M. **C** Venn diagram was employed to provide a comprehensive overview of the characteristics and variations of differential bacteria across all groups

Significant differences in the distribution of GM have been documented between hypertensive patients and HCs. In this study, we elucidated the sex-specific distribution patterns of GM in hypertensive patients through analysis of both alpha and beta diversity metrics. To identify microbial species that exhibited dramatic differences and enrichment at each taxonomic level, we performed a comparative analysis between HTN and HC of single sex. Specifically, we compared the gut GM composition between HTN-F and HC-F, as well as between HTN-M and male HC-M, across various taxonomic levels, including phylum, order, family, genus, and species. The results based on LEfSe analysis revealed a total of 45 differential bacteria distributed across various taxonomic levels between the HTN-F and HC-F groups. Among these, 11 bacterial taxa exhibited an enrichment trend in the HTN group, such as *Hungatella, Lachnoclostridium*, *Clostridium bolteae*, and *Blautia sp. N6H1_15*, whereas 34 differential bacterial taxa demonstrated a reduction trend, including *Lachnospira*, *Roseburia intestinalis*, *Coriobacteriales*, *Bacteroides uniformis*, and *Faecalibacterium* (Fig. 5A). In contrast, a total of 79 differentially abundant bacterial taxa were identified when comparing HTN-M to HC-M. Among these, 26 bacterial taxa were enriched in the HTN group, including *Ruminococcus gnavus*, *Akkermansia*, *Parabacteroides*, and *Butyrivibrio*, whereas 53 bacterial taxa were depleted, such as *Lactobacillus*, *Roseburia intestinalis*, and *Oscillibacter* (Fig. 5B). Notably, when we performed ANCOM-BC2 to filter differential bacterial between HTN-F and HC-F, some features identified in the original LEfSe analysis were validated by ANCOM-BC2 method. The differential bacteria including *Hungatella* in female group and *Ruminococcus gnavus* in male group were also confirmed by this method, and the trend of enrichment or reduction still remained consistent.

Notably, six co-differentiating bacterial taxa were significantly enriched in the HTN group (both females and males), including *Erysipelotrichaceae*, *Erysipelotrichales*, *Erysipelotrichia*, *Erysipelatoclostridium*, *Eisenbergiella*, and *Blautia sp. CAG_257* (Fig. 5C). Additionally, nine shared bacterial species were observed to be significantly reduced in the hypertensive group. These include *Roseburia intestinalis*, *Firmicutes bacterium CAG_110*, *Coprococcus eutactus*, *Coprococcus comes*, *Ruminococcus callidus*, *Ruminococcus champanellensis*, *Gemmiger formicilis*, and *Lactobacillus rogosae* (Fig. 5C). These bacteria, which were both enriched and reduced in the HTN group irrespective of sex, may be closely associated with the disease symptoms of HTN.

Furthermore, it was observed that five specific flora exhibited significant enrichment in HTN-F, a phenomenon that was not evident in HTN-M, including *Lachnoclostridium*, *Clostridium bolteae*, *Hungatella*, *Hungatella hathewayi*, and *Blautia sp. N6H1_15*. 25 bacterial taxa were specifically depleted in HTN-F, including *Mageeibacillus indolicus*, *Eubacterium sp. CAG_251*, *Ruminococcus sp. CAG_330*, *Catenibacterium*, *Faecalibacterium prausnitzii*, *Coriobacteriales*, *Collinsella* (Fig. 5C). And some of these results can also be confirmed by ANCOM-BC2 analysis. For example, *Hungatella* and *Hungatella hathewayi* still exhibited an increased abundance in HTN-F while *Eubacterium sp. CAG_251* and *Ruminococcus sp. CAG_330* showed a lower abundance. The sex-specific variations in these microbial communities may be intricately linked to the pathogenesis of HTN in different sexes.

### Correlation analysis of gut bacteria across different groups

**Fig. 6:**
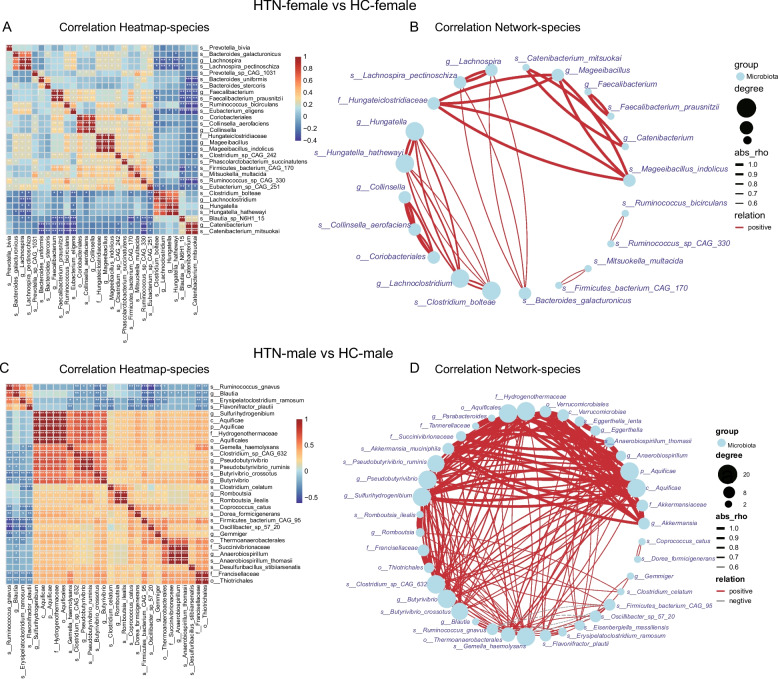
Correlation analysis of differential bacteria between HTN and HC based on sex differences. **A** Heatmap displaying the top 30 differential bacteria in HTN-F and HC-F using Spearman correlation analysis. Different colors represented the magnitude of the correlation coefficients, with red indicating positive correlations and blue indicating negative correlations. *, *p <* 0.05; **, *p <* 0.01; ***, *p <* 0.001, denoting the significance levels of the correlations. **B** Diagram further elucidating the correlation network among the top 30 differential organisms between female HTNs and HCs. The differences were significant (*p <* 0.05), and the correlation coefficients between each pair exceeded 0.5. Red lines were positive correlations between nodes, while blue lines indicated negative correlations. All correlations were based on Spearman correlation analysis. **C** Heatmap illustrating spearman correlation results for the top 30 differential bacteria between HTN-M and HC-M. **D** Network diagram depicting the correlation relationships among the differential bacteria between HTN-M and HC-M. Red and blue connecting lines denoted positive and negative correlations, respectively, and all connections meet the criteria of significance (*p <* 0.05) and absolute correlation coefficients greater than 0.5

In this study, we performed a comprehensive and in-depth correlation analysis comparing the intestinal flora of HTN-F with HC-F, as well as HTN-M with HC-M. Figure 6A illustrates the correlation between the distinct intestinal microbiota in hypertensive female patients and healthy female controls. Our analysis revealed significant correlations among specific bacterial species within the female cohort, including *Lachnospira pectinoschiza*, *Bacteroides stercoris*, and *Faecalibacterium prausnitzii*. The bacterial species relationship network further elucidates the interconnections between various bacterial species in the GM of HTN-F and HC-F. The results indicated the presence of specific bacterial species within the intestinal flora network of HTN-F, including *Hungatella hathewayi*, *Collinsella aerofaciens*, and *Mageeibacillus indolicus*. These species exhibited a high degree of connectivity within the network, suggesting that they may play crucial roles in shaping the structure of the intestinal microbiota (Fig. 6B). The analysis of the correlation between the gut flora of HTN-M and HC-M revealed significant associations among certain bacterial species, including *Erysipelatoclostridium*, *Blautia*, and *Flavonifractor plautii*, with other taxa (Fig. 6C). In males, *Eggerthella lenta*, *Gemella haemolysans*, and *Anaerobiospirillum thomasii* exhibited a high degree of connectivity within the network (Fig. 6D). The correlation of those differential bacteria confirmed by both LEfSe and ANCOM-BC2 methods were further demonstrated by heatmaps and network diagrams in Figure S1. Furthermore, male species exhibited more pronounced connectivity, while inter-species associations in females were less robust.

**Fig. 7:**
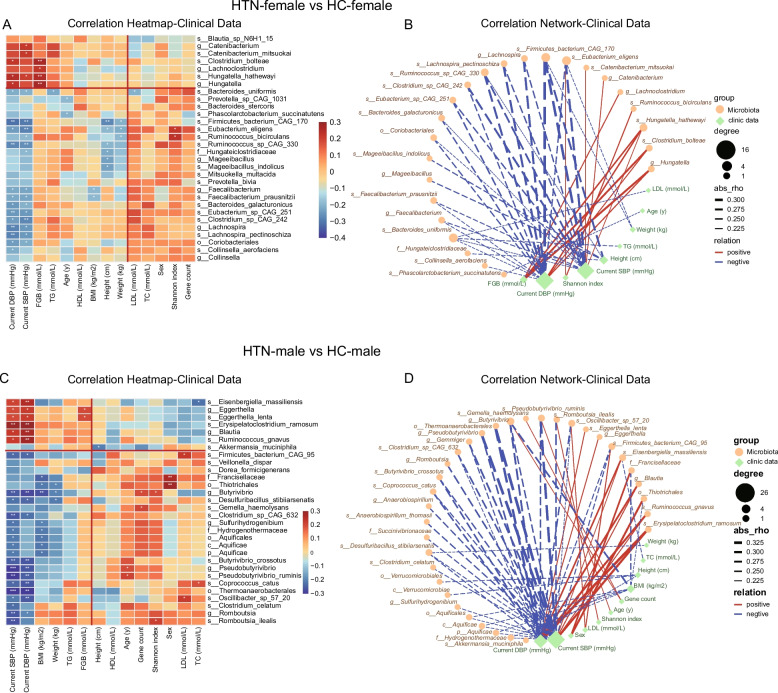
The correlations between bacteria in HTN and HC groups, as stratified by sex, and their respective clinical characteristics. **A** A heat map illustrated the correlation of the top 30 differentially abundant bacteria between HTN-F and HC-F, along with each participant's clinical characteristics, based on Spearman correlation analysis. The color gradient reflected the magnitude of the correlation coefficients, where red denotes positive correlations and blue indicates negative correlations. Statistical significance was marked as follows: *, *p <* 0.05; **, *p <* 0.01; ***, *p <* 0.001. **B** The network diagram further depicted the correlation between the top 30 differentially abundant bacteria in HTN-F and HC-F and the clinical characteristics. Significant differences between groups were indicated by correlation coefficients exceeding 0.2. Red lines signified positive correlations, while blue lines denoted negative correlations. Orange circles represented differentially abundant organisms, and green diamonds represented clinical features. This diagram elucidated the intricate relationships between differential organisms and clinical features in the female population. **C** A Spearman correlation heat map for the top 30 differentially abundant bacteria between HTN-M and HC-M and each participant's clinical characteristics was presented. **D** The network diagram illustrated the correlation between the top 30 differentially abundant bacteria in HTN-M and HC-M and their clinical characteristics. This diagram provided insight into the complex associations between differential bacteria and clinical characteristics in hypertensive male patients compared to healthy male controls

To assess the relationship between differential gut bacteria and the clinical characteristics of the cohort, and explore potential mechanisms underlying their interactions, we employed Spearman correlation analysis. We examined the key clinical characteristics and laboratory test results, including participants' sex, age, SBP, DBP, and lipid profiles, in relation to differential bacteria. The results demonstrated significant correlations between GM and both SBP and DBP. The results were validated through the further identification of intergroup differential bacteria via the ANCOM-BC2 method—an approach additionally employed to complement the LEfSe analysis (Figure S2). Specifically, the differential bacteria observed when comparing HTN-F and HC-F, exhibited significant positive or negative correlations with either SBP or DBP (Fig. 7 A-B).

For instance, when comparing the HTN-F and HC-F groups, *Hungatella*, *Clostridium bolteae*, and *Hungatella hathewayi* exhibited a positive correlation with DBP. In contrast, *Firmicutes bacterium_CAG_170*, *Lachnospira*, *Lachnospira pectinoschiza*, *Ruminococcus sp. CAG_330*, *Clostridium sp. CAG_242*, *Eubacterium sp. CAG_251*, *Bacteroides galacturonicus* and *Coriobacteriales* exhibited significant negative correlations with DBP (Fig. 7 A-B). Furthermore, SBP exhibited a significant negative correlation with *Pseudobutyrivibrio ruminis*, *Thermoanaerobacterales*, *Pseudobutyrivibrio*, and *Romboutsia*, which were differentially abundant between the HTN-M and HC-M groups (Fig. 7 C-D). These findings offer evidence to warrant further investigation into the potential association between HTN and sex.

## Discussion

Substantial sex disparities in hypertensive disorders have been documented, spanning epidemiological characteristics, pathophysiological mechanisms, comorbidities and complications, adverse effects of antihypertensive medications, and prognostic outcomes [6, 8, 32, 33]. However, our understanding of the etiology and underlying mechanisms remains incomplete. Numerous studies have established significant associations between the GM, including its metabolites, and HTN. And comparatively limited research has explored the potential interactions among sex, GM, and HTN. Previous studies have reported that the influence of ACE2 on blood pressure, mediated through the GM-tryptophan-indole pathway, is unique to males [34]. Our study explores sex-related differences in the GM among hypertensive patients using metagenomic approach, seeking to offer new insights into the sex-specific characteristics of HTN. We identified a statistically significant difference in microbial diversity when comparing the GM of hypertensive individuals and HCs upon stratification of the HTN and HC groups by sex. When further analysis was performed in sex subgroups, variations in gut flora distribution were observed in females by PCA analysis, and GM structural characteristics were comparatively more similar in males. Additionally, several representative genera of gut flora, including *Lachnoclostridium*, *Lachnospira*, *Roseburia intestinalis*, *Coriobacteriales*, *Bacteroides uniformis*, and *Faecalibacterium*, exhibited significant differences when comparing HTN females to healthy control females. These results provide a potential direction for understanding the heightened susceptibility to hypertensive organ damage in female hypertensive patients and contributes to advanced understanding the relationship between sex and GM.

It was identified in the present work that *Lachnoclostridium* exhibited a pronounced trend of enrichment in HTN-F, which is consistent with the findings of Moira D and colleagues, who also reported a positive correlation between *Lachnoclostridium* and SBP [35]. Furthermore, a study conducted by other researchers reported a significantly higher abundance of *Lachnoclostridium* in individuals with elevated blood pressure, which aligns with our observations [36]. Notably, *Lachnoclostridium* is significantly enriched in the GM of patients with cardiovascular diseases, such as atrial fibrillation and atherosclerosis. This enrichment leads to elevated serum trimethylamine N-oxide levels, which in turn contributes to accelerated plaque formation in vivo [37, 38]. What’s more, *Lachnoclostridium* also exhibited a significant and positive correlation with both triglycerides and cholesterol levels [39]. Therefore, the genus *Lachnoclostridium*, which is significantly enriched in female hypertensive patients, may contribute to elevated blood pressure by increasing serum trimethylamine N-oxide concentrations.

In contrast, the abundance of *Lachnospira* was significantly diminished in HTN-F. Research has demonstrated that a reduction in *Lachnospira* within the intestinal tract is correlated with various diseases, including asthma in children, pediatric Crohn's disease, colorectal cancer concurrent with inflammatory bowel disease, and chronic kidney disease [40–44]. Besides *Roseburia intestinalis*, which we observed to be significantly reduced in HTN-F, has also been demonstrated to exhibit a decreasing trend in diseases such as inflammatory bowel disease, type 2 diabetes, antiphospholipid syndrome, and atherosclerosis [45, 46]. Moreover, it has been previously documented that the abundance of *Roseburia* is suppressed in both hypertensive patients and animal models [25, 47]. As an important butyrate-producing gut bacterium, *Roseburia intestinalis* was confirmed to effectively inhibit inflammation and mitigate atherosclerosis in mice through its metabolite, butyric acid [48]. The predominant gut-associated butyrate-producing bacterial species, *Roseburia intestinalis*, interacts with dietary phytopolysaccharides to modulate intestinal gene expression, which redirects metabolic pathways from glycolysis towards fatty acid utilization, thereby reducing systemic inflammation and consequently ameliorating cardiovascular injuries [48]. Based on these studies, it is speculated that *Lachnospira* may exhibit a negative correlation with inflammatory factors, while *Roseburia intestinalis* is capable to enhance metabolic processes and inhibit the formation of atherosclerotic plaques. The diminished abundance of *Lachnospira* and *Roseburia intestinalis* in hypertensive women may contribute to the onset, progression, and exacerbation of target organ damage associated with HTN.

*Bacteroides uniformis* was found to be significantly depleted in HTN-F. Previous studies have shown that *Bacteroides uniformis* exhibits a negative correlation with visceral fat accumulation and is associated with the folate synthesis pathway [49]. Additionally, it has been demonstrated to reduce hepatic steatosis as well as hepatic cholesterol and triglyceride concentrations in mice [50]. Furthermore, *Bacteroides uniformis* was identified to play a pivotal role in modulating oral glucose tolerance, enhancing hepatic glucose storage, and selectively influencing intestinal lipid absorption as well as serum triglyceride levels [51]. *Bacteroides uniformis* is known to mitigate obesity and enhance glucose homeostasis by maintaining intestinal immune homeostasis in obese patients, which is partly attributed to increased butyrate production, and reduced systemic inflammatory markers [52]. *Faecalibacterium*, one of the most abundant bacterial genera in the human gastrointestinal microbiota, is a significant producer of butyrate [53]. Notable reductions in *Faecalibacterium* abundance have been observed in patients with gout, cirrhosis, and type 2 diabetes, and the inhibition of butyrate biosynthesis in these conditions exhibits striking similarities [53–56]. We observed a significant reduction in the relative abundance of both *Faecalibacterium* and *Coprococcus* in HTN-F. Interestingly, *Coprococcus* is also the most important butyrate-producing bacteria [57, 58].

Based on our findings, it was observed that the genera *Lachnospira*, *Faecalibacterium*, *Roseburia* exhibited negative correlation with blood pressure. Notably, the abundance of these differentially abundant bacteria was reduced in HTN-F subjects and they were primarily associated with the biosynthesis of SCFAs, particularly butyrate. Several studies have previously demonstrated that producers of SCFAs, including members of the *Roseburia*, are associated with reduced blood pressure [59]. Circulating SCFAs can influence host physiology through multiple mechanisms, including serving as ligands for G protein-coupled receptors to modulate host blood pressure [60, 61]. A randomized controlled trial conducted by Chen et al. revealed that moderate sodium reduction significantly elevated circulating levels of SCFAs, including butyrate, isobutyrate, and valerate, in untreated hypertensive patients [62]. Given that SCFAs are gut microbial metabolites and virtually all circulating SCFAs originate from microbial activity [63], this finding supports the notion that sodium reduction modulates GM structure, thereby altering the host's SCFA metabolic profile. Animal studies have demonstrated that high-salt diets decrease certain bacteria abundance and butyrate production, whereas sodium restriction might reverse these effects [64, 65]. Notably, sex-stratified analysis showed that female hypertensive patients exhibited more pronounced increases in SCFAs (e.g., isobutyrate, isovalerate, 2-methylbutyrate) following sodium restriction, and these changes were inversely correlated with reductions in systolic blood pressure (SBP), diastolic blood pressure (DBP), and 24-h ambulatory blood pressure [62]. These findings align with animal experiments demonstrating that SCFAs lower blood pressure by activating G protein-coupled receptors (e.g., Gpr41) or olfactory receptors (Olfr78) [66, 67]. Regarding the sex-specific effects of sodium restriction on SCFAs, the researchers observed more significant SCFAs responses and stronger associations with blood pressure reductions in female patients [62]. Therefore, the aberrant alterations in the abundance of these differentially abundant bacteria in hypertensive female patients result in changes in their associated metabolite SCFAs, which may subsequently contribute to the progression and exacerbation of HTN in these patients.

Despite the lack of significant differences in the distribution of GM between the HTN-M and HC-M groups, we conducted analysis of the microbial flora between these two groups. The results indicated that bacteria such as *Ruminococcus gnavus*, *Akkermansia*, and *Parabacteroides* were enriched in the HTN-M group, whereas genera including *Lactobacillus* and *Roseburia* were significantly depleted. The abundance of *Ruminococcus gnavus* was reported to be significantly elevated in patients with inflammatory bowel diseases, including Crohn's disease of the small intestine [68, 69]. Additionally, *Ruminococcus gnavus* was shown to exhibit a positive correlation with indicators of insulin resistance in patients concurrently diagnosed with irritable bowel syndrome and diabetes mellitus [55]. In our study, *Ruminococcus gnavus* was significantly enriched in the HTN-M group and exhibited a notable positive correlation with both systolic and diastolic blood pressure. In contrast, the abundance of *Lactobacillus* was dramatically reduced in HTN-M. The down-regulation of *Lactobacillus*, a probiotic known for its anti-inflammatory properties, has been extensively linked to various diseases, including irritable bowel syndrome, inflammatory bowel disease, type I diabetes mellitus, and colorectal cancer [70]. Research has indicated that patients with multiple sclerosis and children with allergies exhibit reduced or absent levels of *Lactobacillus spp.* in their intestinal flora. Studies have also demonstrated a negative correlation between *Lactobacillus* and pro-inflammatory immune markers, as well as a positive correlation with anti-inflammatory immune markers [71]. Based on our findings and the previous researches, there maybe a potential pro-inflammatory implications of *Ruminococcus gnavus* enrichment [56] and a lack of anti-inflammatory effects of *Lactobacillus* depletion [59] in male hypertensive patients. These changes may represent a distinct microbial signature in males, thereby mediating mechanisms different from those of females. It was indicated that differential bacteria in male hypertensive patients exhibited more pronounced pro-inflammatory immune characteristics.

A recent cross-sectional clinical trail conducted by Lv et al. revealed that the abundance of some SCFAs-producting related microbiota was reduced in hypertensive females, indicating the aberrant abundance alternations may further lead to the progression and exacerbation of HTN [72]. SCFAs can be produced by gut microbiome and have sex-specific implications due to the different production levels in females or males [73]. The findings of our study are consistent with the previous report in terms of female-specific dysbiosis in HTN. In addition, male ACE2-deficient rats displayed heightened inflammatory markers in the intestine and exhibited an increase in *Escherichia/Shigella* abundance, which were widely considered as metabolize tryptophan, whereas these changes were not observed at the genus level in females [34]. Bardhan et al. contributed the novel direction to the blood pressure regulation landscape by uncovering sex-specific differences in tryptophan metabolism through the GM mediating [74]. These findings support the notion that differential bacteria in male hypertensive patients exhibited more pronounced pro-inflammatory immune characteristics. Another Asian cohort confirmed that significant differences in β-diversity and GM composition between HTN patients and health control group were only observed in female but not in male [24]. This is concordant with our findings of sex-specific association between gut microbiome and HTN, indicating that GM dysregulation appears to be more strongly associated with HTN in female.

This study revealed significant sex differences in HTN-related GM dysbiosis, providing important insights for clinical translation. The reduction of butyrate-producing bacteria such as *Lachnospira*, *Faecalibacterium*, and *Roseburia* in female hypertensive patients, as well as the enrichment of *Ruminococcus gnavus* in males, could serve as sex-specific biomarkers to aid in HTN risk assessment. For interventions, females may benefit from supplementation with these butyrate-producing bacteria or high-fiber diets to promote their proliferation, leveraging butyrate’s vasoregulatory and anti-inflammatory effects to improve blood pressure and target organ damage. In males, targeted elimination strategies for *Ruminococcus gnavus* could be explored, alongside supplementation of anti-inflammatory *Lactobacillus*. Future randomized controlled trials, considering sex-specific traits, are needed to validate the efficacy of these strategies, facilitating precise HTN management.

However, there are several limitations that warrant consideration. Firstly, the sample size of this study is relatively limited, which may have a certain impact on the power of statistical tests. For the analysis of beta diversity in the male population, we did not observe significant differences. However, considering the sample size of the male subgroup, the possibility of Type II error cannot be completely ruled out, and the truly existing differences in beta diversity may not have been fully detected due to insufficient sample size. This suggests that a larger sample size may be crucial for revealing potential differences in sex-specific microbial community characteristics. Given the above limitations, this study is regarded as an exploratory analysis with its core value lies in preliminarily revealing sex differences in the gut microbiome of essential HTN, thereby providing testable hypotheses for subsequent research. Future studies need to be validated in larger independent cohorts. In addition, combining multi-center data or more refined stratified analyses (e.g., stratification by HTN duration or medication status) may further clarify the association between sex-specific microbiota characteristics and HTN, and enhance the robustness of the conclusions. Secondly, the relationship between gut microbial metabolites, metabolic pathways, and blood pressure requires more in-depth exploration. Additionally, mechanistic studies utilizing animal models are essential to elucidate the causality and role of commensal bacteria in the sex-specific pathophysiology of HTN.

In summary, an imbalance in the intestinal microbiota appears to be more strongly associated with female HTN patients. Bacteria such as *Lachnospira*, *Faecalibacterium*, and *Roseburia*, which exhibit a negative correlation with blood pressure, show a marked reduction among female hypertensive patients. Meanwhile, some specific microbial alterations are also observed between male hypertensive patients and normal controls, with the bacteria exhibited favoring pro-inflammatory immune characteristics. The aberrant SCFAs-producing flora in female hypertensive patients may contribute to higher risk of hypertensive organ damage observed in women compared to men. Sex should therefore be considered as an important factor when evaluating the role of GM in HTN.

## Supplementary Information

Supplementary Material 1: Figure S1. Association of differential GM in HTNs and HCs in female and male, respectively. (A) The correlation between differential bacteria between HTN-F and HC-F was examined by Spearman correlation analysis. Positive correlation was depicted in red and negative correlation was in blue. *, *p*<0.05; **, *p*<0.01; ***, *p*<0.001. (B) Correlation network (red line, positive correlation; blue line, negative correlation) among differential organisms between female HTNs and HCs, based on Spearman correlation analysis. The differences were significant (p<0.05), and the correlation coefficients between each pair exceeded 0.2. (C) The spearman correlation results for the differential bacteria between HTN-M and HC-M were demonstrated by heatmap. (D) Network illustrating the correlation relationship among the differential bacteria between male HTNs and HCs. All significantly differential bacteria in female or male were the overlap between differential abundance testing results of LEfSe and ANCOM-BC2. Figure S2. The correlations between differential bacteria and clinical phenotypes in HTN and HC groups, as clustered by sex. (A) Heatmap demonstrating the Spearman correlation analysis results of the significantly differential bacteria and clinical characteristics between HTN-F and HC-F. Positive association was in red color while negative was in blue color. *, *p*<0.05; **, *p*<0.01; ***, *p*<0.001. (B) Network exhibiting the correlation of differential bacteria in female group and clinical phenotypes. Green circle denoted bacteria, and orange rhombus represented clinicle characteristics. Size of circles or rhombus denoted the degree according to the number of variances connected. The absolute correlation coefficient was greater than 0.2, and p-value was lower than 0.05, as calculated with Spearman correlation. The thickess of lines connecting variants represented the correlation coefficient. (C) Heatmap depicting Spearman rank correlation between differential bacterial in HTN-M and HC-M and clinical indexes. (D) Association network showed correlations between clinical data and bacteria distinct in HTN-M and HC-M. The significantly differential bacteria in the above Spearman analysis in female or male were attributable to the intersection of differential abundance testing results from both LEfSe and ANCOM-BC2.

Supplementary Material 2.

## Data Availability

The dataset supporting the results of this study is archived from the European Bioinformatics Institute database under the accession number ERP023883.

## Abbreviations

HTN: Hypertension
GM: Gut microbiota
SCFAs: Short-chain fatty acids
