# Supplementary material for: A validation for sex differences in gut microbiome of essential hypertension based on cohort analysis
Source: BMC Microbiol. 2026 Feb 7;26:255. doi: 10.1186/s12866-025-04500-8 (PMC13001194; doi:10.1186/s12866-025-04500-8)
Supplement: Supplementary file 1 — Supplementary Material 1: Figure S1. Association of differential GM in HTNs and HCs in female and male, respectively. (A) The correlation between differential bacteria between HTN-F and HC-F was examined by Spearman correlation analysis. Positive correlation was depicted in red and negative correlation was in blue. *, p<0.05; **, p<0.01; ***, p<0.001. (B) Correlation network (red line, positive correlation; blue line, negative correlation) among differential organisms between female HTNs and HCs, based on Spearman correlation analysis. The differences were significant (p<0.05), and the correlation coefficients between each pair exceeded 0.2. (C) The spearman correlation results for the differential bacteria between HTN-M and HC-M were demonstrated by heatmap. (D) Network illustrating the correlation relationship among the differential bacteria between male HTNs and HCs. All significantly differential bacteria in female or male were the overlap between differential abundance testing results of LEfSe and ANCOM-BC2. Figure S2. The correlations between differential bacteria and clinical phenotypes in HTN and HC groups, as clustered by sex. (A) Heatmap demonstrating the Spearman correlation analysis results of the significantly differential bacteria and clinical characteristics between HTN-F and HC-F. Positive association was in red color while negative was in blue color. *, p<0.05; **, p<0.01; ***, p<0.001. (B) Network exhibiting the correlation of differential bacteria in female group and clinical phenotypes. Green circle denoted bacteria, and orange rhombus represented clinicle characteristics. Size of circles or rhombus denoted the degree according to the number of variances connected. The absolute correlation coefficient was greater than 0.2, and p-value was lower than 0.05, as calculated with Spearman correlation. The thickess of lines connecting variants represented the correlation coefficient. (C) Heatmap depicting Spearman rank correlation between differential b [file 12866_2025_4500_MOESM1_ESM.zip › Figure-S2.pdf]

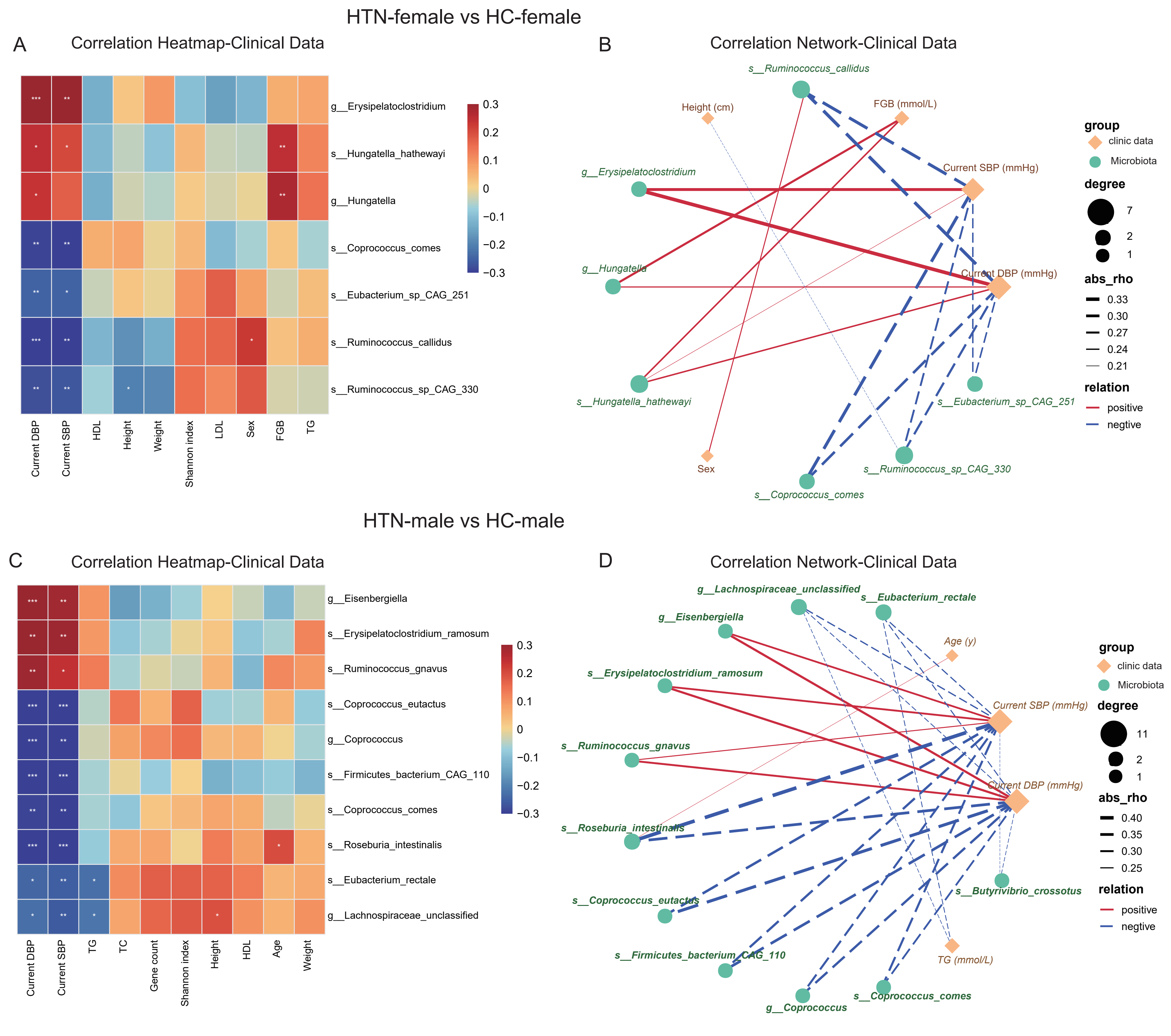
HTN-female vs HC-female
A
Correlation Heatmap-Clinical Data
g__Erysipelatoclostridium
s__Hungatella_hathewayi
g__Hungatella
s__Coprococcus_comes
s__Eubacterium_sp_CAG_251
s__Ruminococcus_callidus
s__Ruminococcus_sp_CAG_330
Current DBP
Current SBP
HDL
Height
Weight
Shannon index
LDL
Sex
FGB
TG
0.3
0.2
0.1
0
−0.1
−0.2
−0.3
B
Correlation Network-Clinical Data
s__Ruminococcus_callidus
Height (cm)
FGB (mmol/L)
g__Erysipelatoclostridium
Current SBP (mmHg)
g__Hungatella
Current DBP (mmHg)
s__Hungatella_hathewayi
s__Eubacterium_sp_CAG_251
Sex
s__Ruminococcus_sp_CAG_330
s__Coprococcus_comes
group
clinic data
Microbiota
degree
7
2
1
abs_rho
0.33
0.30
0.27
0.24
0.21
relation
positive
negtive
HTN-male vs HC-male
C
Correlation Heatmap-Clinical Data
g__Eisenbergiella
s__Erysipelatoclostridium_ramosum
s__Ruminococcus_gnavus
s__Coprococcus_eutactus
g__Coprococcus
s__Firmicutes_bacterium_CAG_110
s__Coprococcus_comes
s__Roseburia_intestinalis
s__Eubacterium_rectale
g__Lachnospiraceae_unclassified
Current DBP
Current SBP
TG
TC
Gene count
Shannon index
Height
HDL
Age
Weight
0.3
0.2
0.1
0
−0.1
−0.2
−0.3
D
Correlation Network-Clinical Data
g__Lachnospiraceae_unclassified
s__Eubacterium_rectale
g__Eisenbergiella
Age (y)
s__Erysipelatoclostridium_ramosum
Current SBP (mmHg)
s__Ruminococcus_gnavus
Current DBP (mmHg)
s__Roseburia_intestinalis
s__Butyrivibrio_crossotus
s__Coprococcus_eutactus
s__Firmicutes_bacterium_CAG_110
TG (mmol/L)
g__Coprococcus
s__Coprococcus_comes
group
clinic data
Microbiota
degree
11
2
1
abs_rho
0.40
0.35
0.30
0.25
relation
positive
negtive
