# Supplementary figures and images for: A validation for sex differences in gut microbiome of essential hypertension based on cohort analysis
Source: BMC Microbiol. 2026 Feb 7;26:255. doi: 10.1186/s12866-025-04500-8 (PMC13001194; doi:10.1186/s12866-025-04500-8)

## HTN-female vs HC-female

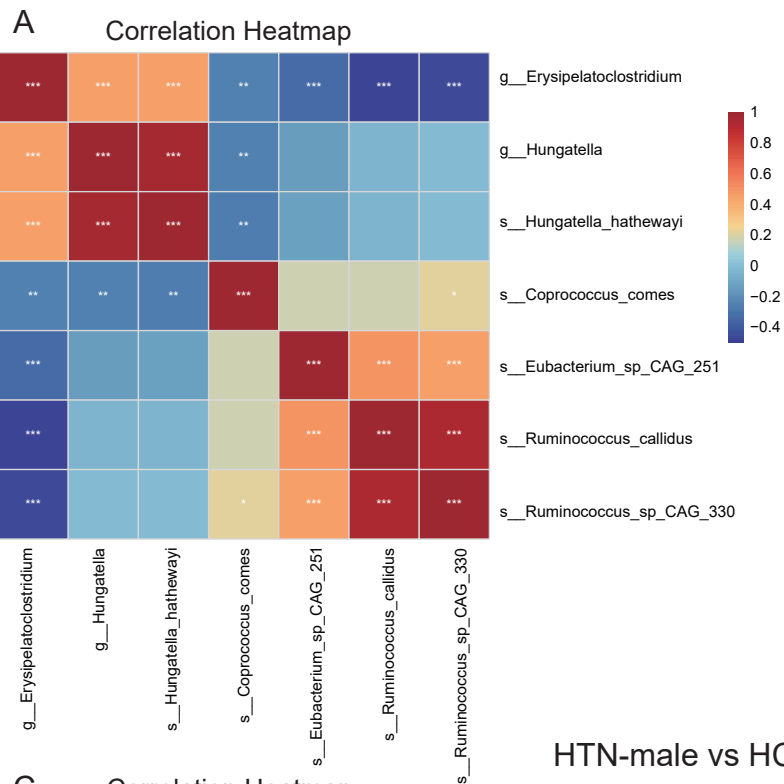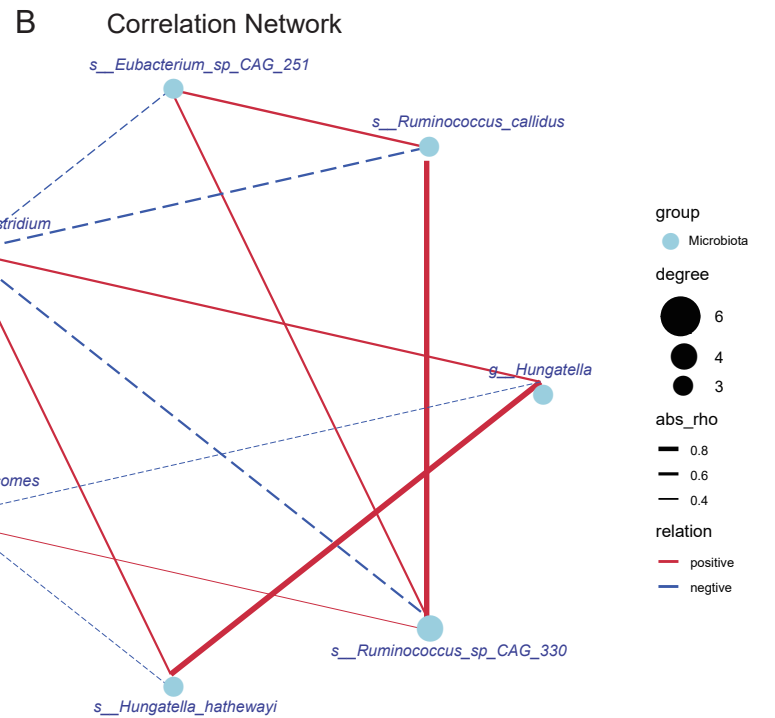

## HTN-male vs HC-male

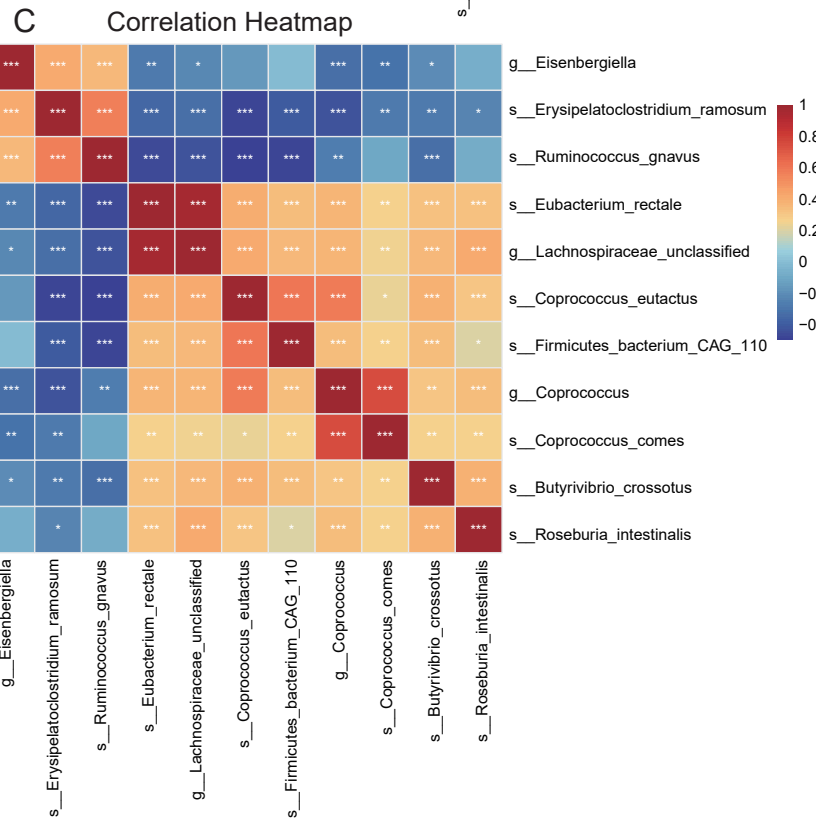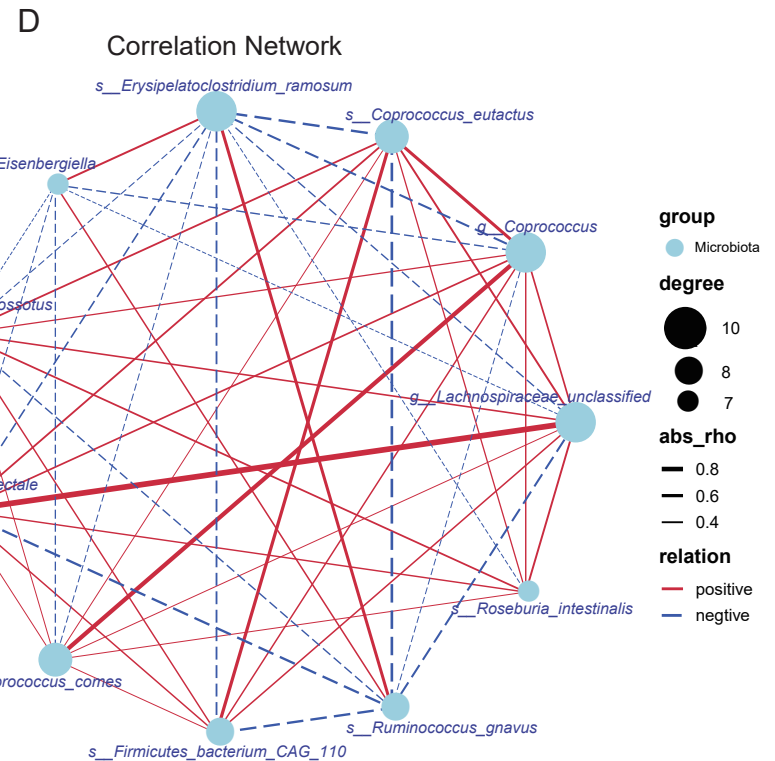

Supplement: Supplementary file 1 — Supplementary Material 1: Figure S1. Association of differential GM in HTNs and HCs in female and male, respectively. (A) The correlation between differential bacteria between HTN-F and HC-F was examined by Spearman correlation analysis. Positive correlation was depicted in red and negative correlation was in blue. *, p<0.05; **, p<0.01; ***, p<0.001. (B) Correlation network (red line, positive correlation; blue line, negative correlation) among differential organisms between female HTNs and HCs, based on Spearman correlation analysis. The differences were significant (p<0.05), and the correlation coefficients between each pair exceeded 0.2. (C) The spearman correlation results for the differential bacteria between HTN-M and HC-M were demonstrated by heatmap. (D) Network illustrating the correlation relationship among the differential bacteria between male HTNs and HCs. All significantly differential bacteria in female or male were the overlap between differential abundance testing results of LEfSe and ANCOM-BC2. Figure S2. The correlations between differential bacteria and clinical phenotypes in HTN and HC groups, as clustered by sex. (A) Heatmap demonstrating the Spearman correlation analysis results of the significantly differential bacteria and clinical characteristics between HTN-F and HC-F. Positive association was in red color while negative was in blue color. *, p<0.05; **, p<0.01; ***, p<0.001. (B) Network exhibiting the correlation of differential bacteria in female group and clinical phenotypes. Green circle denoted bacteria, and orange rhombus represented clinicle characteristics. Size of circles or rhombus denoted the degree according to the number of variances connected. The absolute correlation coefficient was greater than 0.2, and p-value was lower than 0.05, as calculated with Spearman correlation. The thickess of lines connecting variants represented the correlation coefficient. (C) Heatmap depicting Spearman rank correlation between differential b [file 12866_2025_4500_MOESM1_ESM.zip › Figure-S1.pdf]
